# Psoriasis in a patient with dermatomyositis and allergic contact dermatitis to rubber: A case report

**DOI:** 10.1016/j.jdcr.2026.09.026

**Published:** 2026-09-23

**Authors:** Donn LaTour, Iman Ali, Sara Zoghi, Nada Mohamed, Shannon C. Brown

**Affiliations:** a Department of Dermatology, Baylor Scott & White Medical Center, Texas A&M University College of Medicine, Temple, Texas; b Department of Dermatology, Baylor College of Medicine, Temple, Texas; c Department of Internal Medicine, Texas Health Presbyterian Dallas, Dallas, Texas; d Texas A&M University Naresh K. Vashisht College of Medicine, Bryan, Texas

**Keywords:** allergy, anti-MDA5 dermatomyositis, contact dermatitis, dermatomyositis, pustular psoriasis

## Introduction

Psoriasis and dermatomyositis are chronic, systemic inflammatory diseases that rarely coexist. Psoriasis is a common inflammatory dermatosis associated with multiple comorbidities, including psoriatic arthritis, cardiometabolic disease, and depression.^1^ Co-occurrence with connective tissue diseases is rare, reported in only 0.69% of cases.^2^ Histologically, psoriasis demonstrates parakeratosis, hyperkeratosis, elongation of rete ridges, thinning of the suprapapillary epidermis, and loss of stratum granulosum. In pustular psoriasis, dense neutrophilic infiltrates form spongiform pustules and microabscesses.^3^

Dermatomyositis is an autoimmune disease characterized by inflammation of the muscles and skin, with several clinical variants.^4^ Clinically amyopathic dermatomyositis (CADM) presents with classic cutaneous findings without objective muscle involvement. The CADM-5 subtype is associated with anti-MDA5 antibodies. Histopathologic findings include epidermal atrophy, basal vacuolar change, basement membrane degeneration, sparse perivascular lymphocytic infiltrates, and interstitial mucin deposition.^3^

Notably, psoriasis and dermatomyositis share inflammatory pathways including, interleukin (IL)-17, type 1 interferons (IFN-α/β), and tumor necrosis factor (TNF)-α.^3^ Recognition of this overlap is clinically significant given the elevated malignancy risk associated with dermatomyositis and the therapeutic challenges posed by overlapping disease activity.^4^ Systemic corticosteroids can exacerbate psoriasis, while biologic agents for psoriasis may worsen dermatomyositis. We present a case of new-onset pustular psoriasis in a patient with CADM-5 dermatomyositis and allergic contact dermatitis (ACD). This case primarily illustrates how treating one disease can unmask or exacerbate the other, as suggested here by the emergence of psoriasis following corticosteroid withdrawal. The accompanying diagnostic nuance and shared immunologic pathways further explain why this overlap is difficult to manage.

## Case report

A 72-year-old female presented with a 6-month history of a progressive rash beginning on her right arm and spreading to the chest, face, and scalp, associated with photosensitive burning and proximal muscle fatigue. She worked in a shoe-repair shop with frequent rubber exposure and had patch testing positive (2+) for black rubber mix.

Examination revealed violaceous to erythematous papules coalescing into plaques in a shawl distribution, periungual erythema with dilated capillary loops, ragged cuticles, and painful erythematous palmar plaques. An outside punch biopsy showed interface dermatitis with basement membrane thickening, consistent with connective tissue disease.

Laboratory evaluation revealed mildly elevated erythrocyte sedimentation rate (21 mm/hr) and aspartate aminotransferase (46 U/L), with normal creatine kinase and aldolase. A myositis panel was positive for anti-MDA5 antibodies. Total-body positron emission tomography was negative for malignancy, and chest computed tomography revealed nonspecific patchy ground-glass opacities with a stable 7-mm left upper lobe nodule, supporting a diagnosis of anti-MDA5 dermatomyositis.

Initial treatment with topical corticosteroids, hydroxychloroquine, and a prednisone taper resulted in partial improvement. Hydroxychloroquine was discontinued due to suspected drug reaction. She was transitioned to intravenous immunoglobulin (IVIG), which achieved sustained dermatomyositis control. Courses of systemic corticosteroids provided transient benefit with rebound flares.

The patient subsequently experienced multiple hospital admissions for diffuse eruptions. Examination revealed well-demarcated erythematous patches with collarettes of scale and scattered pustules, most prominent in intertriginous areas (Fig 1, *A*-*C*). Punch biopsies from contact and non-contact sites with the rubber wheelchair armrests were obtained to assess the contribution of contact allergy. Both showed subcorneal pustules with scattered eosinophils and psoriasiform hyperplasia, without interface dermatitis, dermal mucin, spongiosis, or Langerhans cell microabscesses (Fig 2, *A* and *B*). The identical findings at non-contact sites indicated that the eruption was not localized to areas of rubber exposure, favoring psoriasis exacerbated by Koebnerization rather than primary ACD.

**Fig 1:**
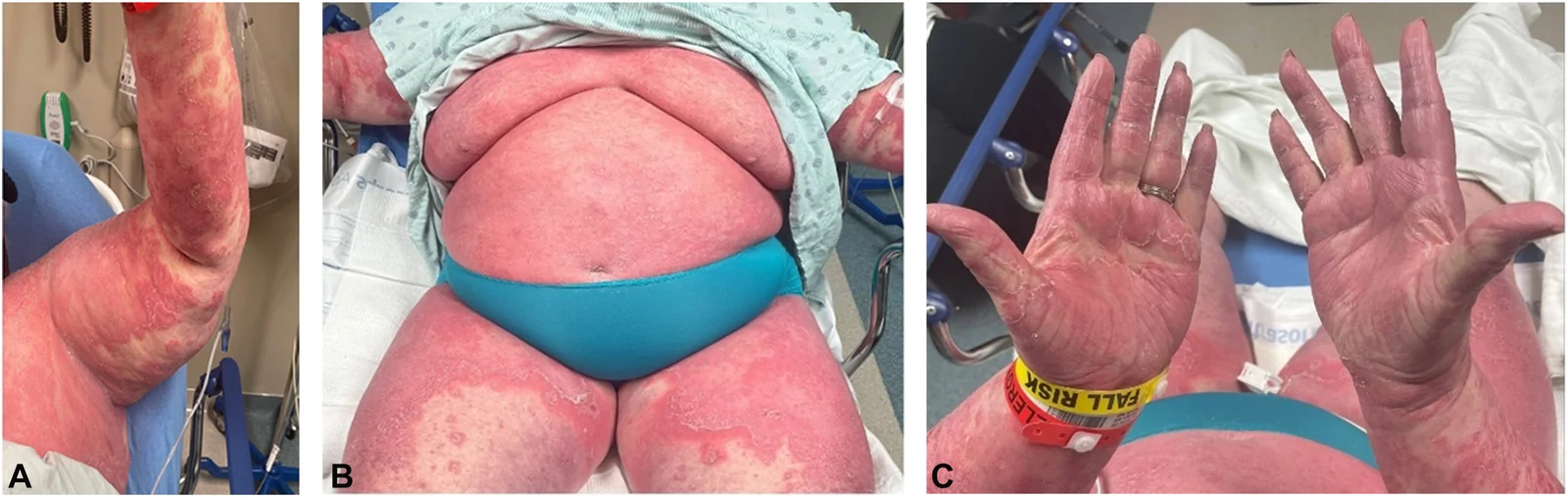
**A,** Scaly red confluent plaques with scattered pustules on the left forearm, arm, and lateral trunk consistent with patient’s psoriasis. **B,** Desquamating, red, scaly plaques bordering on erythroderma and confluent over the breasts, abdomen, and upper thighs consistent with patient’s psoriasis. **C,** Desquamating red scaly plaques involving the bilateral palms consistent with patient’s psoriasis.

**Fig 2:**
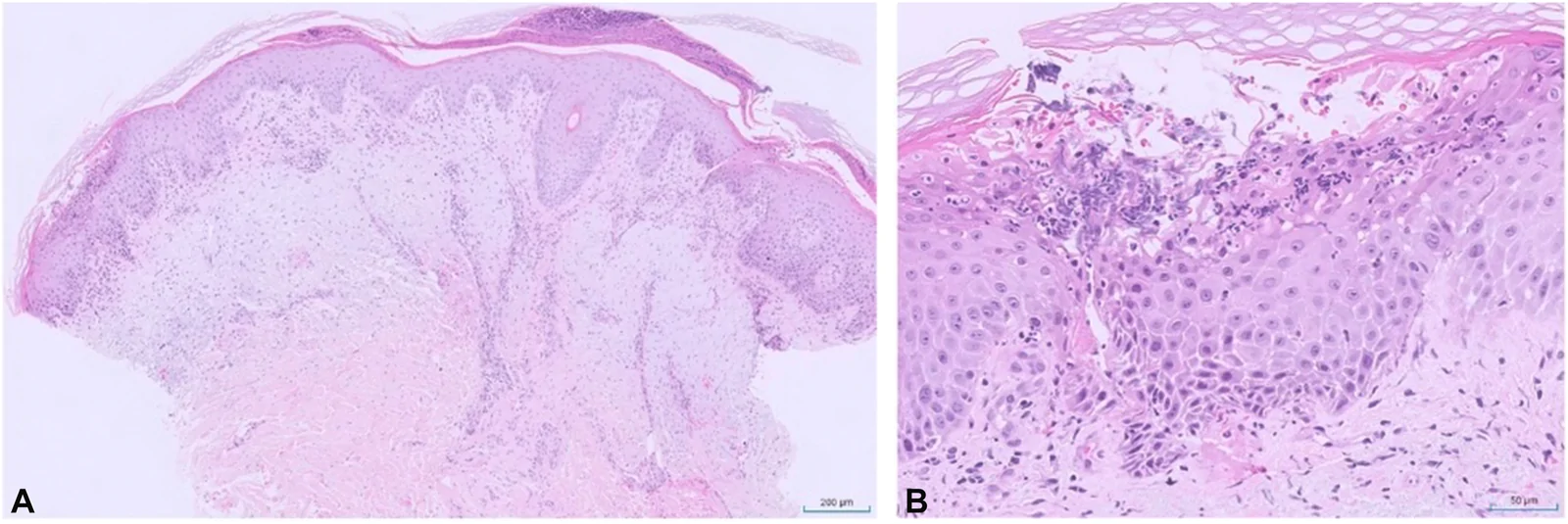
**A,** Low-power view showing mild epidermal hyperplasia with a subcorneal pustule and overlying parakeratosis containing collections of neutrophils (H&E, ×50). **B,** High-power view showing a subcorneal pustule with an interstitial mixed inflammatory infiltrate composed of neutrophils, histiocytes, lymphocytes, and rare eosinophils (H&E, ×400).

Five months after her dermatomyositis diagnosis, the eruption evolved into classic plaque and inverse psoriasis involving the trunk, inframammary folds, and gluteal cleft. Treatment with a prednisone taper and risankizumab led to marked improvement.

Following IVIG discontinuation, she developed a dermatomyositis flare with painful erythematous papules on the palms and violaceous papules and plaques on the scalp, face, and chest (Fig 3, *A* and *B*). Repeat biopsy (Fig 4, *A*-*C*) demonstrated interface dermatitis with necrotic keratinocytes and mild dermal mucin. She was restarted on IVIG and transitioned to rituximab, achieving dermatomyositis control while maintaining psoriasis remission on risankizumab.

**Fig 3:**
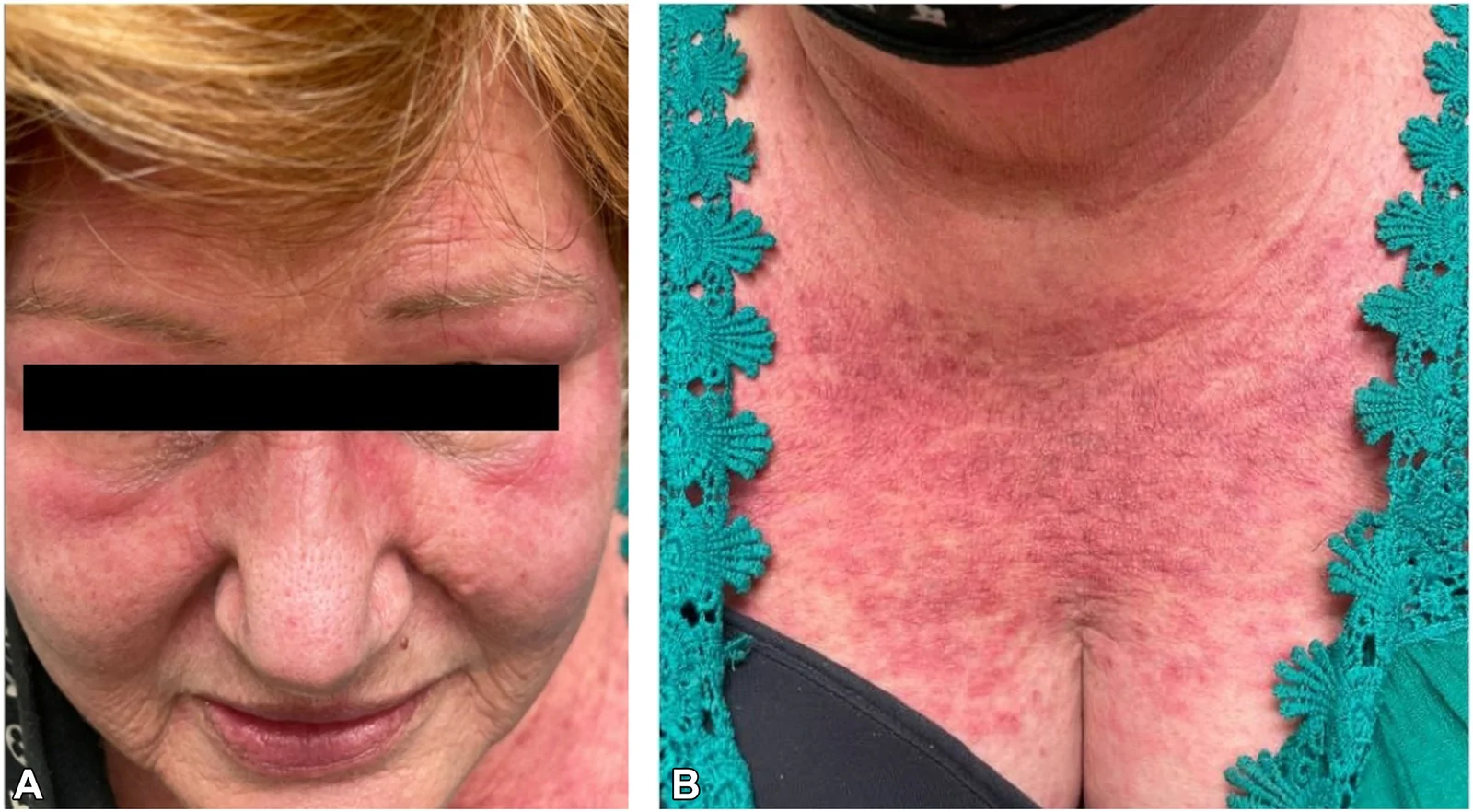
**A,** Red to purple patches involving the forehead, upper eyelids, and bilateral cheeks and nasal root consistent with patient’s dermatomyositis. **B,** Pink to violet plaques over the upper chest consistent with patient’s dermatomyositis.

**Fig 4:**
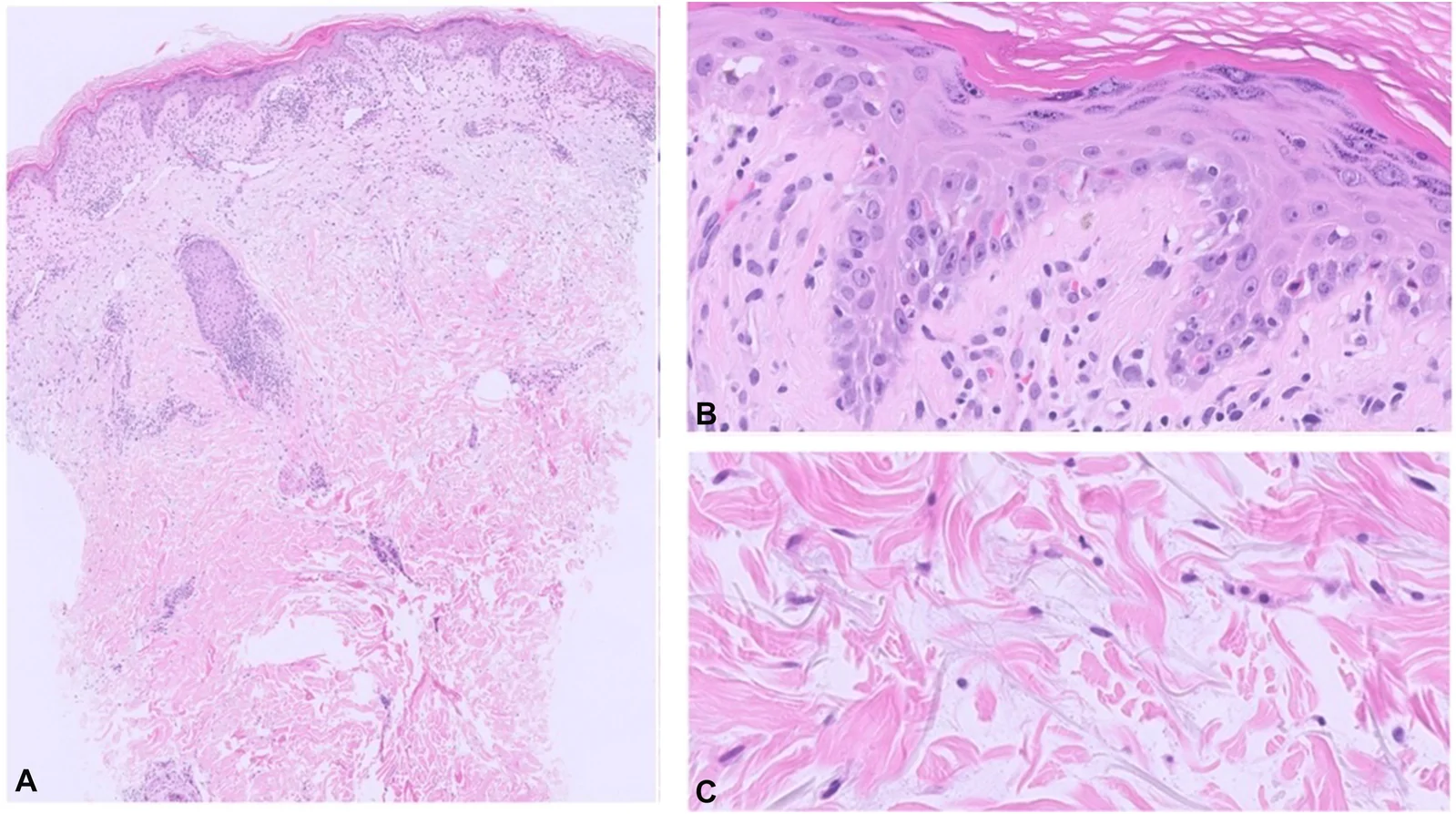
**A,** Low-power view showing interface changes and a sparse perivascular infiltrate composed predominantly of lymphocytes (H&E, ×20). **B,** Demonstrates vacuolar interface changes with basal cell vacuolization and necrotic keratinocytes at the dermoepidermal junction (H&E, ×400). **C,** Demonstrates mild dermal mucin deposition (H&E, ×400).

## Discussion

The patient’s evolving, morphologically distinct eruption brought into question several competing diagnoses, including dermatomyositis, psoriasis, ACD, and AGEP. Punch biopsy did not favor a diagnosis of dermatomyositis, as the absence of interface dermatitis and dermal mucin argued against it. Moreover, the previously-diagnosed dermatomyositis lesions appeared violaceous and photodistributed with periungual and palmar involvement, whereas the new eruption was scaly and pustular, favoring intertriginous sites. Thus, the findings suggested other diagnoses. Dermal eosinophils initially raised concern for AGEP or ACD; however, eosinophils are frequently observed in both psoriasis and dermatomyositis and should not exclude either diagnosis. In psoriasis, counts exceeding approximately 5 eosinophils per high-power field are more suggestive of alternative diagnoses.^5^ Eosinophils have also been described in dermatomyositis, where they may correlate with chronic inflammation rather than a distinct eosinophilic dermatosis.^6^ Our patient’s modest eosinophilic infiltrate is compatible with either disease process and likely reflects overlapping inflammatory pathways rather than a third, unrelated diagnosis. Although biopsies were taken from areas corresponding to wheelchair contact, they lacked spongiosis or Langerhans cell microabscesses, features that favor ACD when present.^7^ The documented rubber allergy contributed primarily to diagnostic confusion, raising ACD on the differential, as opposed to serving as the primary driver of the eruption. AGEP was excluded clinically by eliminating candidate exposure medications and careful follow-up. While the positive patch test confirms contact sensitivity, the distribution and histology argue against it as the main process. Rather, the rubber exposure may have acted as a Koebnerizing stimulus in the setting of systemic immune dysregulation. Taken together, the subcorneal pustules and psoriasiform hyperplasia, the exclusion of AGEP and ACD, and the eruption's subsequent evolution into classic plaque and inverse psoriasis supported a diagnosis of psoriasis.

Beyond diagnosis, this case highlights the challenges of treating inflammatory diseases. Systemic corticosteroids, although effective for dermatomyositis, can precipitate psoriasis during tapering.^3^ Steroid-sparing agents such as IVIG, rituximab, or targeted biologics may be preferable. IL-23 inhibitors, including risankizumab, can effectively control psoriasis while generally avoiding dermatomyositis exacerbation, whereas antimalarials and TNF- α inhibitors should be used cautiously due to reports of psoriasiform flares and dermatomyositis induction.^8^

Repeated corticosteroid withdrawal may have contributed to the unmasking of psoriasis in this patient, a sequence described in prior reports of dermatomyositis-associated psoriasis.^9^ The absence of a personal or family history of psoriasis and the temporal relationship to dermatomyositis treatment support an acquired, therapy-triggered process.

Psoriasis preceded dermatomyositis in 6 of approximately 15 reported cases.^9^ Evidence guiding treatment of coexisting dermatomyositis and psoriasis remains limited and derives entirely from individual case reports. In 1 reported case, cyclosporine achieved disease control after failure of multiple immunomodulatory therapies, but no study has compared treatments in patients with both diseases.^9^^,^^10^ In our patient, psoriasis emerged during treatment of established dermatomyositis, a sequence in which corticosteroid withdrawal has been reported as a trigger in a case.^9^ More research is needed to determine the optimal management strategies, ideally through multicenter case series or registry-based analyses.

Clinicians should remain vigilant for malignancy in patients with dermatomyositis, particularly when the disease presents atypically, and ensure appropriate age- and risk-adjusted cancer screening. In summary, this case demonstrates how management of coexisting dermatomyositis and psoriasis is complicated when therapy for 1 disease exacerbates the other, most notably when corticosteroid withdrawal may have unmasked psoriasis. It emphasizes cautious corticosteroid use and thoughtful immunomodulatory selection, guided by careful diagnostic distinction between the 2 processes.

## Conflicts of interest

None disclosed.
